# Prevalence of Underlying Medical Conditions Among Selected Essential Critical Infrastructure Workers — Behavioral Risk Factor Surveillance System, 31 States, 2017–2018

**DOI:** 10.15585/mmwr.mm6936a3

**Published:** 2020-09-11

**Authors:** Sharon R. Silver, Jia Li, Winifred L. Boal, Taylor L. Shockey, Matthew R. Groenewold

**Affiliations:** 1 Division of Field Studies and Engineering, National Institute for Occupational Safety and Health, CDC.

Certain underlying medical conditions are associated with higher risks for severe morbidity and mortality from coronavirus disease 2019 (COVID-19) (*1*). Prevalence of these underlying conditions among workers differs by industry and occupation. Many essential workers, who hold jobs critical to the continued function of infrastructure operations (*2*), have high potential for exposure to SARS-CoV-2, the virus that causes COVID-19, because their jobs require close contact with patients, the general public, or coworkers. To assess the baseline prevalence of underlying conditions among workers in six essential occupations and seven essential industries, CDC analyzed data from the 2017 and 2018 Behavioral Risk Factor Surveillance System (BRFSS) surveys, the most recent data available.* This report presents unadjusted prevalences and adjusted prevalence ratios (aPRs) for selected underlying conditions. Among workers in the home health aide occupation and the nursing home/rehabilitation industry, aPRs were significantly elevated for the largest number of conditions. Extra efforts to minimize exposure risk and prevent and treat underlying conditions are warranted to protect workers whose jobs increase their risk for exposure to SARS-CoV-2.

BRFSS is an annual, state-based, random-digit–dialed landline and cellular telephone survey collecting demographic and health-related information among noninstitutionalized U.S residents aged ≥18 years. BRFSS includes standard core questions and optional modules, including an industry and occupation module. All participants are asked to report their height and weight and also asked “Has a doctor, nurse, or other health practitioner ever told you that you have…” followed by a list of underlying conditions.^†^ In 2017 and 2018, 31 states^§^ administered the industry and occupation module for at least 1 year to currently or recently employed participants; the study sample comprised currently employed module respondents.^¶^ Open-ended responses to questions eliciting respondent industry and occupation** were coded to the U.S. Census Bureau’s 2010 industry and occupation codes.^††^ Among states using the industry and occupation questions, the median overall survey response rate was 42.5% in 2017 ^§§^ and 49.1% in 2018. ^¶¶^

Respondent demographic characteristics, as well as weighted, unadjusted prevalences and aPRs for selected underlying conditions, were obtained for a subset of critical infrastructure worker groups selected because of their inability to work from home or physically distance from others at work, and their potential exposure to infectious disease (*3*,*4*), as well as adequate sample size in the data set.*** Six occupation groups were selected: 1) health practitioners (licensed health care professionals except technicians/technologists), 2) health technicians and technologists, 3) other health care support (except home health), 4) patient and personal care aides in the home health industry (home health aides), 5) protective services (correctional officers, police, sheriffs, patrol officers, firefighters, and their supervisors), and 6) teachers (preschool through grade 12). Seven industry groups were selected: 1) ambulatory health care, 2) hospitals, 3) nursing homes (nursing and residential care facilities), 4) essential retail (grocery/other food stores, alcohol stores, pharmacies, and gas stations), 5) food manufacturing, 6) transit (bus service/urban transit, taxi/limousine, postal services, and couriers/messengers), and 7) trucking. Health conditions from the BRFSS core module with strong or mixed evidence of associations with severe outcomes from COVID-19 that were evaluated included asthma (current, ever diagnosed), cancer (except nonmelanoma skin cancer), coronary heart disease (CHD; myocardial infarction, angina, or coronary heart disease), chronic kidney disease, chronic obstructive pulmonary disease (COPD), diabetes, hypertension, obesity (body mass index [BMI] ≥30 kg/m^2^, calculated from respondent’s self-reported height and weight), severe obesity (BMI ≥40 kg/m^2^), and stroke. Hypertension questions were asked only in 2017.

For each occupation or industry, demographic distributions and unadjusted prevalences for each chronic condition were calculated using the SURVEYFREQ procedure in SAS (version 9.4; SAS Institute). Logistic regression in SUDAAN (version 11.0.1; RTI International) was used to calculate aPRs to compare the prevalence of each condition in the occupation (or industry) of interest to its prevalence among workers from all other U.S. Census-coded occupations (or industries), essential and nonessential combined, except the group of interest. Adjustments were made for age group (18–29, 30–39, 40–49, 50–59, 60–69, ≥70 years), sex (male, female), and race/ethnicity (non-Hispanic White, Black, Asian, or other race, and Hispanic). aPRs with confidence intervals not spanning the null value were considered statistically significant. Data were weighted and analyzed in accordance with the survey’s complex sampling design.

The study population comprised 213,518 respondents meeting the analytic criteria (Table 1). At least 15% (weighted percentage) of workers in the health practitioner and home health aide occupations and the ambulatory health care, transit, and trucking industries were aged ≥60 years. Males comprised 85.2% of protective service workers and 89.2% of trucking industry workers. At least 25% of home health aide occupation, nursing home industry, and transit industry workers were non-Hispanic Black. The percentages of Hispanic workers were highest in the home health aide occupation (20%) and the food manufacturing industry (36%).

**TABLE 1:** Demographic characteristics of selected essential workers, by industries (I) and occupations (O) — Behavioral Risk Factor Surveillance System, 31 U.S. states,* 2017–2018

Worker grouping^†^	Total respondents	No. (%)^§^										
		Age (yrs)				Sex		Race/Ethnicity^¶^				
		18–49	50–59	60–69	≥70	Male	Female	White	Black	Asian	Other	Hispanic
All workers**	213,518	110,540 (66.0)	54,423 (20.8)	37,596 (10.6)	10,959 (2.7)	109,590 (54.9)	103,654 (45.1)	157,236 (59.4)	18,088 (12.1)	7,406 (6.5)	10,934 (3.0)	19,854 (19.0)
Occupation												
Health practitioners O = 3000–3260	12,208	5,943 (60.4)	3,245 (23.7)	2,519 (13.6)	501 (2.3)	2,703 (22.8)	9,496 (77.2)	9,884 (66.7)	910 (11.5)	506 (11.4)	410 (1.9)	498 (8.5)
Health technicians and technologists O = 3300–3535	3,164	1,760 (67.5)	794 (20.5)	534 (10.1)	76 (1.9)	730 (29.6)	2,428 (70.4)	2,429 (68.8)	297 (12.2)	104 (9.4)	151 (2.7)	183 (6.9)
Health care support (except home health) O = 3600–3655, excluding I = 8170	3,368	2,144 (76.5)	697 (14.5)	452 (8.0)	75 (1.0)	368 (11.0)	2,997 (89.0)	2,073 (50.7)	626 (24.2)	123 (4.9)	193 (3.5)	353 (16.7)
Home health patient and personal care aides O = 3600 or 4610, restricted to I = 8170	1,179	531 (59.4)	328 (23.2)	236 (13.4)	84 (4.0)	139 (10.2)	1,040 (89.8)	644 (39.0)	253 (30.6)	35 (5.3)	92 (4.9)	155 (20.2)
Protective services O = 3700–3720, 3740–3750, 3800–3860	2,422	1,618 (73.5)	584 (21.1)	194 (4.5)	26 (0.9)	1,950 (85.2)	471 (14.8)	1,780 (66.4)	267 (15.3)	38 (1.8)	171 (3.9)	166 (12.6)
Teachers O = 2300–2330	8,965	4,741 (66.6)	2,468 (22.5)	1,478 (9.5)	278 (1.5)	1,934 (23.4)	7,020 (76.6)	7,215 (71.6)	708 (12.7)	191 (3.7)	392 (2.5)	459 (9.5)
Industry												
Ambulatory health care I = 7970–8090	9,679	4,853 (63.3)	2,444 (21.2)	1,900 (13.0)	482 (2.6)	2,580 (28.5)	7,091 (71.5)	7,424 (63.3)	817 (12.8)	413 (10.6)	460 (2.4)	565 (11.0)
Hospitals I = 8190	12,155	6347 (64.1)	3,293 (22.3)	2,175 (11.8)	340 (1.8)	3,000 (27.5)	9,138 (72.5)	8,994 (61.0)	1,339 (15.2)	509 (9.5)	527 (2.6)	786 (11.7)
Nursing homes and rehabilitation I = 8270, 8290	3,833	1903 (61.9)	973 (23.9)	762 (11.6)	195 (2.5)	566 (18.4)	3,266 (81.6)	2,621 (54.9)	717 (27.6)	97 (4.8)	162 (2.4)	236 (10.4)
Essential retail I = 4970–4990, 5070, 5090	4,399	2,432 (72.2)	998 (16.0)	745 (9.5)	224 (2.4)	2,021 (54.0)	2,372 (46.0)	3,232 (60.3)	304 (8.6)	194 (7.3)	268 (4.0)	401 (19.8)
Food manufacturing I = 1070–1370	1,682	954 (71.6)	443 (17.5)	238 (8.0)	47 (3.0)	1,037 (63.7)	642 (36.3)	987 (44.4)	171 (11.4)	35 (5.1)	73 (3.2)	416 (35.9)
Transit, postal, messengers, and couriers I = 6180, 6190, 6370, 6380	1,932	836 (61.6)	599 (23.2)	388 (13.0)	109 (2.2)	1,205 (67.5)	723 (32.5)	1,225 (47.0)	339 (25.6)	79 (6.7)	122 (4.6)	167 (16.2)
Trucking I = 6170	2,418	1,134 (59.4)	718 (23.6)	447 (14.2)	119 (2.8)	2,109 (89.2)	307 (10.8)	1,729 (55.3)	345 (22.5)	41 (3.3)	123 (3.7)	180 (15.1)

* California, Connecticut, Delaware, Florida, Georgia, Hawaii, Illinois, Indiana, Kansas, Louisiana, Maryland, Massachusetts, Michigan, Minnesota, Mississippi, Missouri, Montana, Nebraska, New Hampshire, New Jersey, New Mexico, New York, North Carolina, North Dakota, Pennsylvania, Rhode Island, South Carolina, Tennessee, Texas, Washington, and Wisconsin.

^†^ By U.S. Census codes (https://www.census.gov/programs-surveys/cps/technical-documentation/methodology/industry-and-occupation-classification.html).

^§^ Weighted percentage.

^¶^ White, Black, and Asian are non-Hispanic. Other are respondents identifying as non-Hispanic and not identifying specifically as Asian, Black, or White. Respondents identifying with multiple races are asked to select the grouping that best represents their race.

** All currently employed non-active duty military respondents to the Industry and Occupation module of the 2017 or 2018 Behavioral Risk Factor Surveillance System.

Prevalences of preexisting underlying conditions varied by occupation (Table 2) and industry (Table 3). Obesity and hypertension were the most common conditions in every essential worker group. Among occupations, home health aides had the highest unadjusted prevalence estimate for every chronic condition except severe obesity and had significantly elevated aPRs for five conditions (chronic kidney disease, COPD, diabetes, obesity, and severe obesity,). For health care support workers (other than home health), aPRs were significantly elevated for diabetes, obesity, and severe obesity. In contrast, among health practitioners, aPRs for many conditions were significantly below 1.0. Among workers in the nursing home industry, aPRs for CHD, COPD, diabetes, hypertension, obesity, and severe obesity were significantly elevated. Non-health care industries with statistically significant elevations in aPRs for more than one underlying condition included transit (current asthma and diabetes) and trucking (COPD, obesity, and severe obesity).

**TABLE 2:** Prevalence* and adjusted prevalence ratio (aPR)^†^ of underlying health conditions among essential workers, by occupation^§^ — Behavioral Risk Factor Surveillance System, 31 U.S. states,^¶^ 2017–2018

Underlying condition	All workers**	Health practitioners	Health technicians and technologists	Health care support (except home health)	Home health and personal care aides	Protective services	Teachers, pre-K–grade 12
Asthma, current							
% (95% CI)	7.6 (7.4–7.9)	10.0 (8.7–11.5)	9.3 (7.2–11.7)	10.3 (8.5–12.4)	13.2 (9.6–17.6)	6.9 (5.0–9.2)	11.4 (9.8–13.2)
aPR (95% CI)	—	1.08 (0.94–1.25)	0.99 (0.78–1.27)	0.98 (0.80–1.19)	1.31 (0.96–1.78)	1.04 (0.78–1.39)	1.19 (1.02–1.39)
Asthma, ever							
% (95% CI)	12.8 (12.4–13.1)	14.4 (12.7–16.1)	14.6 (11.6–18.2)	14.3 (12.2– 16.7)	17.1 (12.9–22.0)	13.6 (11.0–16.5)	16.6 (14.6–18.8)
aPR (95% CI)	—	1.04 (0.92–1.18)	1.02 (0.81–1.28)	0.90 (0.76–1.06)	1.16 (0.88–1.78)	1.11 (0.92–1.35)	1.17 (1.03–1.33)
Cancer^††^							
% (95% CI)	3.7 (3.5–3.8)	4.0 (3.5–4.7)	3.5 (2.7–4.6))	3.0 (1.9–4.4)	5.0 (3.2–7.4)	2.6 (1.6–3.9)	4.3 (3.4–5.3)
aPR (95% CI)	—	0.84 (0.72–0.98)	0.85 (0.65–1.12	0.83 (0.57–1.22)	1.02 (0.68–1.54)	0.96 (0.64–1.44)	0.96 (0.78–1.19)
Coronary heart disease^§§^							
% (95% CI)	3.0 (2.8–3.2)	2.0 (1.5–2.6)	1.4 (1.0–2.0)	2.2 (1.5–3.2)	4.4 (2.0–8.3)^¶¶^	2.7 (1.5–4.5)	1.6 (1.1–2.3)
aPR (95% CI)	—	0.75 (0.57–0.99)	0.64 (0.45–0.90)	1.32 (0.92–1.89)	1.80 (0.93–3.45)	0.95 (0.57–1.57)	0.70 (0.48–1.01)
Chronic kidney disease							
% (95% CI)	1.6 (1.5–1.7)	1.3 (1.0–1.7)	1.6 (0.8–2.9)^¶¶^	1.0 (0.5–1.6)	4.6 (2.0–9.0)^¶¶^	1.6 (0.8–3.0)^¶¶^	1.4 (1.0–1.9)
aPR (95% CI)	—	0.79 (0.59–1.05)	1.07 (0.58–2.00)	0.65 (0.37–1.12)	2.53 (1.24–5.14)	1.22 (0.66–2.26)	0.90 (0.64–1.27)
COPD							
% (95% CI)	3.1 (2.9–3.2)	1.7 (1.4–2.1)	3.0 (2.0–4.3)	4.0 (2.9–5.4)	6.2 (4.0–9.0)	2.5 (1.1–4.7)^¶¶^	2.7 (1.8–3.8)
aPR (95% CI)	—	0.46 (0.37–0.57)	0.91 (0.63–1.30)	1.25 (0.92–1.70)	1.68 (1.14–2.48)	0.89 (0.46–1.71)	0.76 (0.53–1.08)
Diabetes							
% (95% CI)	6.5 (6.3–6.8)	5.6 (4.7–6.5)	5.9 (4.5–7.5)	6.6 (5.2–8.1)	12.2 (8.2–17.4)	7.1 (5.0–9.7)	5.4 (3.9–7.3)
aPR (95% CI)	—	0.85 (0.72–1.00)	1.02 (0.80–1.31)	1.36 (1.10–1.67)	1.70 (1.21–2.39)	1.13 (0.83–1.53)	0.93 (0.69–1.25)
Hypertension***							
% (95% CI)	23.7 (23.1–24.4)	20.3 (18.1–22.6)	23.2 (18.8–28.2)	21.2 (17.1–25.7)	29.3 (22.4–37.1)	25.6 (20.4–31.3)	17.8 (15.4–20.4)
aPR (95% CI)	—	0.86 (0.78–0.96)	1.11 (0.94–1.31)	1.10 (0.94–1.30)	1.15 (0.89–1.48)	1.04 (0.86–1.26)	0.81 (0.72–0.92)
Obesity (BMI≥30 kg/m^2^)^†††^							
% (95% CI)	29.9 (29.4–30.4)	26.1 (23.7–28.5)	37.4 (32.7–42.3)	40.0 (36.6–43.5)	44.8 (36.9–53.0)	39.6 (35.7–43.6)	27.3 (25.1–29.7)
aPR (95% CI)	—	0.86 (0.78–0.93)	1.27 (1.12–1.45)	1.29 (1.19–1.41)	1.38 (1.12–1.69)	1.24 (1.12–1.37)	0.86 (0.79–0.94)
Severe obesity (BMI≥40 kg/m^2^)^†††^							
% (95% CI)	4.3 (4.1–4.5)	3.3 (2.7–4.1)	4.1 (3.0–5.6)	9.1 (7.2–11.2)	9.1 (6.0–13.0)	5.5 (3.6–8.0)	4.9 (3.8–6.3)
aPR (95% CI)	—	0.67 (0.54–0.82)	0.86 (0.64–1.16)	1.62 (1.29–2.03)	1.59 (1.09–2.31)	1.26 (0.86–1.86)	0.95 (0.73–1.23)
Stroke							
% (95% CI)	1.2 (1.1–1.3)	0.8 (0.5–1.1)	1.7 (0.5–4.2)^¶¶^	0.9 (0.5–1.5)	2.0 (0.8–3.9)^¶¶^	0.3 (0.1–0.7)^¶¶^	1.3 (0.6–2.4)^¶¶^
aPR (95% CI)	—	0.67 (0.47–0.95)	1.68 (0.66–4.29)	0.99 (0.60–1.65)	1.50 (0.74–3.09)	0.32 (0.16–0.66)	1.23 (0.67–2.26)

**Abbreviations:** BMI = body mass index; CI = confidence interval; COPD = chronic obstructive pulmonary disease.

* Unadjusted, weighted estimates.

^†^ Adjusted for age group (18–29, 30–39, 40–49, 50–59, 60–69, ≥70 years), sex, race/ethnicity (non-Hispanic White, non-Hispanic Black, non-Hispanic Asian, non-Hispanic other race, Hispanic). aPR reference group is all other occupations (essential and non-essential) combined.

^§^ By U.S. Census codes (https://www.census.gov/programs-surveys/cps/technical-documentation/methodology/industry-and-occupation-classification.html).

^¶^ California, Connecticut, Delaware, Florida, Georgia, Hawaii, Illinois, Indiana, Kansas, Louisiana, Maryland, Massachusetts, Michigan, Minnesota, Mississippi, Missouri, Montana, Nebraska, New Hampshire, New Jersey, New Mexico, New York, North Carolina, North Dakota, Pennsylvania, Rhode Island, South Carolina, Tennessee, Texas, Washington, and Wisconsin.

** All currently employed non-active duty military respondents to the Industry and Occupation module of the 2017 or 2018 Behavioral Risk Factor Surveillance System.

^††^ Except nonmelanoma skin cancer.

^§§^ Includes heart attack/myocardial infarction, coronary heart disease, or angina.

^¶¶^ Relative standard error >30% but ≤50%.

*** 2017 BRFSS data only, available for 22 states: California, Connecticut, Florida, Georgia, Hawaii, Illinois, Louisiana, Massachusetts, Michigan, Minnesota, Mississippi, Montana, Nebraska, New Hampshire, New Jersey, New Mexico, New York, North Carolina, North Dakota, Tennessee, Washington, and Wisconsin.

^†††^ Body mass index (and thus obesity) was missing for 9% of cohort; all other behaviors and conditions missing for <1% of cohort.

**TABLE 3:** Prevalence* and adjusted prevalence ratio (aPR)^†^ of underlying health conditions among essential workers by industry^§^ — Behavioral Risk Factor Surveillance System, 31 U.S. states,^¶^ 2017–2018

Underlying condition	All workers**	Ambulatory health care	Hospitals	Nursing homes and rehabilitation	Essential retail	Food manufacturing	Transit	Trucking
Asthma, current								
% (95% CI)	7.6 (7.4–7.9)	9.7 (8.4–11.1)	9.7 (8.4–11.0)	10.1 (8.3–12.0)	9.8 (7.7–12.3)	4.6 (2.7–7.4)	11.1 (7.2–16.0)	4.3 (3.0–5.9)
aPR (95% CI)	—	1.07 (0.93–1.24)	1.06 (0.92–1.21)	1.00 (0.83–1.20)	1.22 (0.97–1.53)	0.65 (0.41–1.04)	1.52 (1.05–2.20)	0.68 (0.50–0.94)
Asthma, ever								
% (95% CI)	12.8 (12.4–13.1)	15.1 (13.4–17.0)	14.6 (12.9–16.5)	15.0 (12.7–17.6)	16.1 (13.5–19.0)	9.1 (6.4–12.3)	13.9 (10.0–18.7)	9.5 (7.3–12.1)
aPR (95% CI)	—	1.11 (0.98–1.25)	1.05 (0.93–1.19)	1.01 (0.86–1.20)	1.17 (0.99–1.38)	0.73 (0.54–0.98)	1.13 (0.84–1.51)	0.82 (0.65–1.05)
Cancer^††^								
% (95% CI)	3.7 (3.5–3.8)	4.7 (4.0–5.5)	3.7 (3.0–4.4)	4.6 (3.2–6.3)	3.3 (2.4–4.4)	2.7 (1.7–4.1)	4.0 (2.1–6.8)	2.8 (2.0–3.8)
aPR (95% CI)	—	1.05 (0.89–1.25)	0.84 (0.70–1.02)	0.99 (0.73–1.35)	1.02 (0.77–1.36)	0.86 (0.58–1.28)	1.10 (0.66–1.84)	0.82 (0.60–1.11)
Coronary heart disease^§§^								
% (95% CI)	3.0 (2.8–3.2)	2.7 (1.9–3.7)	2.0 (1.5–2.6)	4.4 (2.8–6.5)	3.0 (2.0–4.4)	4.4 (2.5–7.1)	5.0 (2.5–8.8)	4.2 (3.0–5.8)
aPR (95% CI)	—	1.02 (0.74–1.40)	0.80 (0.62–1.04)	1.90 (1.28–2.82)	1.21 (0.83–1.77)	1.45 (0.93–2.27)	1.49 (0.84–2.66)	1.05 (0.75–1.49)
Chronic kidney disease								
% (95% CI)	1.6 (1.5–1.7)	1.4 (1.1–1.8)	1.3 (0.9–1.8)	1.4 (0.9–2.1)	2.1 (1.1–3.5)	1.1 (0.4–2.5)^¶¶^	1.8 (1.1–2.7)	1.6 (0.9–2.6)
aPR (95% CI)	—	0.87 (0.66–1.15)	0.78 (0.55–1.10)	0.83 (0.54–1.29)	1.40 (0.81–2.42)	0.72 (0.31–1.65)	1.08 (0.71–1.65)	0.99 (0.60–1.64)
COPD								
% (95% CI)	3.1 (2.9–3.2)	2.5 (1.8–3.3)	2.8 (2.2–3.4)	5.1 (3.8–6.6)	3.7 (2.8–4.9)	2.4 (1.2–4.2)	4.0 (2.6–5.7)	5.3 (3.1–8.3)
aPR (95% CI)	—	0.71 (0.53–0.96)	0.80 (0.64–1.01)	1.43 (1.09–1.88)	1.28 (0.98–1.67)	0.84 (0.48–1.47)	1.27 (0.87–1.85)	1.72 (1.09–2.71)
Diabetes								
% (95% CI)	6.5 (6.3–6.8)	6.3 (5.3–7.4)	6.4 (5.5–7.3)	8.3 (6.6–10.2)	6.5 (5.1–8.2)	7.6 (5.0–11.0)	11.4 (8.3–15.0)	11.1 (8.0–14.9)
aPR (95% CI)	—	0.97 (0.82–1.14)	1.01 (0.87–1.17)	1.29 (1.05–1.59)	1.19 (0.96–1.47)	1.14 (0.82–1.60)	1.40 (1.06–1.84)	1.32 (0.97–1.79)
Hypertension***								
% (95% CI)	23.7 (23.1–24.4)	23.3 (20.7–26.1)	20.2 (18.0–22.5)	27.7 (23.2–32.5)	22.7 (18.8–26.9)	26.6 (18.9–35.4)	23.2 (18.0–29.1)	29.6 (24.4–35.1)
aPR (95% CI)	—	1.01 (0.90–1.13)	0.90 (0.81–1.00)	1.24 (1.08–1.43)	1.09 (0.93–1.28)	1.12 (0.89–1.42)	0.85 (0.68–1.05)	1.01 (0.85–1.20)
Obesity (BMI≥30 kg/m^2^)^†††^								
% (95% CI)	29.9 (29.4–30.4)	28.5 (26.4–30.7)	30.4 (28.4–32.6)	37.2 (33.9–40.7)	30.4 (27.3–33.5)	28.0 (22.3–34.2)	29.8 (25.5–34.5)	48.2 (43.4–53.0)
aPR (95% CI)	—	0.95 (0.88–1.02)	1.00 (0.93–1.08)	1.17 (1.07–1.28)	1.06 (0.96–1.16)	0.89 (0.72–1.10)	0.91 (0.78–1.07)	1.50 (1.36–1.66)
Severe obesity (BMI≥40 kg/m^2^)^†††^								
% (95% CI)	4.3 (4.1–4.5)	4.0 (3.3–4.8)	5.3 (4.4–6.3)	7.9 (6.3–9.7)	5.6 (4.2–7.2)	2.6 (1.8–3.8)	4.6 (3.0–6.7)	7.7 (5.4–10.5)
aPR (95% CI)	—	0.84 (0.69–1.03)	1.10 (0.91–1.33)	1.47 (1.19–1.83)	1.36 (1.04–1.76)	0.60 (0.41–0.87)	1.02 (0.70–1.49)	1.93 (1.40–2.65)
Stroke								
% (95% CI)	1.2 (1.1–1.3)	1.1 (0.6–1.8)	1.0 (0.7–1.4)	1.3 (0.9–1.9)	1.0 (0.7–1.4)	0.6 (0.3–1.0)	1.3 (0.6–2.3)^¶¶^	0.9 (0.4–1.8)^¶¶^
aPR (95% CI)	—	0.94 (0.55–1.61)	0.92 (0.66–1.28)	1.13 (0.75–1.69)	1.09 (0.74–1.59)	0.52 (0.30–0.91)	1.02 (0.55–1.90)	0.70 (0.36–1.38)

**Abbreviations:** BMI = body mass index; CI = confidence interval; COPD = chronic obstructive pulmonary disease.

* Unadjusted, weighted estimates.

^†^ Adjusted for age group (18–29, 30–39, 40–49, 50–59, 60–69, ≥70 years), sex, race/ethnicity (non-Hispanic White, non-Hispanic Black, non-Hispanic Asian, non-Hispanic other race, Hispanic). aPR reference group is all other industries (essential and non-essential) combined.

^§^ https://www.census.gov/programs-surveys/cps/technical-documentation/methodology/industry-and-occupation-classification.html.

^¶^ California, Connecticut, Delaware, Florida, Georgia, Hawaii, Illinois, Indiana, Kansas, Louisiana, Maryland, Massachusetts, Michigan, Minnesota, Mississippi, Missouri, Montana, Nebraska, New Hampshire, New Jersey, New Mexico, New York, North Carolina, North Dakota, Pennsylvania, Rhode Island, South Carolina, Tennessee, Texas, Washington, and Wisconsin.

** All currently employed non-active duty military respondents to the Industry and Occupation module of the 2017 or 2018 Behavioral Risk Factor Surveillance System.

^††^ Except nonmelanoma skin cancer.

^§§^ Includes heart attack/myocardial infarction, coronary heart disease, or angina.

^¶¶^ Relative standard error >30% but ≤50%.

*** 2017 BRFSS data only, available for 22 states: California, Connecticut, Florida, Georgia, Hawaii, Illinois, Louisiana, Massachusetts, Michigan, Minnesota, Mississippi, Montana, Nebraska, New Hampshire, New Jersey, New Mexico, New York, North Carolina, North Dakota, Tennessee, Washington, and Wisconsin.

^†††^ Body mass index (and thus obesity) was missing for 9% of cohort; all other behaviors and conditions missing for <1% of cohort.

## Discussion

In this analysis, aPRs for underlying medical conditions were significantly elevated in several groups of essential workers at risk for exposure to SARS-CoV-2. Workers in the home health aide occupations and the nursing home industry are of particular concern because those groups had high prevalences of and significantly elevated aPRs for a number of underlying conditions. In addition to increased occupational exposure risks, some industry and occupation groups had high percentages of demographic groups that have been identified as being at higher risk for severe COVID-19–associated illness (*2*–*4*). For example, the home health aide occupation and the nursing home industry had high concentrations of workers from demographic groups at elevated risk for severe COVID-19 outcomes, such as non-Hispanic Blacks, Hispanics, and older workers. Racial and ethnic minority groups have been subject to long-standing, systemic social inequities that intersect with work-related exposure risks. These include the inability to practice physical distancing at work or to work from home, low wages, lack of paid sick leave, reliance on public or shared transportation, crowded housing, limited access to health care, and the need to hold multiple jobs (*3*,*5*,*6*). Several of these inequities also hinder management of underlying conditions that increase the risk for severe COVID-19 (*7*). These inequities also pertain to groups of essential workers not assessed in this report because of inadequate sample size (e.g., low levels of health care access among food preparers/servers, agricultural workers, and building/grounds maintenance and support, including housekeepers and janitors in health care) (*8*).

The findings in this report are subject to at least eight limitations. First, the industry and occupation module was administered for at least 1 year by 31 states, but the data collected are not nationally representative. Second, the prevalence of some conditions (e.g., immunologic, liver disease, heart failure, neurologic) and genetic polymorphisms not elicited by BRFSS that might affect COVID-19 disease severity could not be assessed. Third, BRFSS does not assess the severity of high blood pressure or asthma or distinguish between Type 1 and Type 2 diabetes, and the CHD category includes conditions that might not worsen COVID-19 outcomes. Fourth, the strength of the association between some conditions included here and severe COVID-19 is not yet known. Fifth, the health information obtained, including BMI, is self-reported (or calculated from self-reported data), lacks clinical confirmation, and is subject to recall and social desirability biases. Sixth, BMI (and thus obesity/severe obesity) was missing for 8% of respondents. Seventh, the survey does not include information on training about or adherence to workplace exposure mitigation^†††^ strategies. Finally, not all workers in each industry or occupation have the same risk for exposure to infectious disease, and not all essential worker industry and occupation groups were evaluated.

In 2017 and 2018, many essential workers had underlying medical conditions, with high prevalences among groups of health care workers at risk for exposure to SARS-CoV-2, including home health aides and nursing home workers. Although health practitioners had low prevalences of the evaluated underlying conditions, some are at increased risk for SARS-CoV-2 exposure during the performance of medical procedures and as a consequence of sustained close contact with their patients.

The Americans with Disabilities Act addresses employment discrimination against workers with disabilities, including disabilities resulting from chronic conditions (*9*). In addition, prioritization of hazard controls and health care access is needed to minimize exposure risks, prevent and address underlying conditions, and ensure access to emerging clinical prevention and treatment measures, so that employees at risk for work-related exposure to SARS-CoV-2 can continue to safely perform their essential workplace functions.

**Summary**

**What is already known about this topic?**

Underlying medical conditions increase risk for severe COVID-19. Many essential workers have high potential for exposure to SARS-CoV-2, the virus that causes COVID-19, because their jobs require close contact with patients, the public, or coworkers.

**What is added by this report?**

High prevalences of underlying medical conditions increase risks for severe COVID-19 illness among home health aides, other health care support workers, and nursing home, trucking, and transit industry workers.

**What are the implications for public health practice?**

For all essential workers, and particularly those at high risk because of underlying medical conditions, prioritization of exposure controls and health care access is needed to reduce the potential for SARS-CoV-2 exposure and prevent and treat underlying conditions.

## Footnotes

* BRFSS collects information on demographics and health, including underlying conditions, use of preventive services, health care access, and health-related behavioral risk factors. https://www.cdc.gov/brfss/index.html.

† Health conditions were elicited by the question “Has a doctor, nurse, or other health practitioner ever told you that you have…” followed by a set of conditions, including those used in this analysis: a heart attack, also called a myocardial infarction; angina or coronary heart disease; stroke; asthma (with positive responses followed by “Do you still have asthma?”); any other type of cancer (other than skin cancer); chronic obstructive pulmonary disease, emphysema, or chronic bronchitis; kidney disease (not including kidney stones, bladder infection, or incontinence); diabetes (followed by questions allowing separation of gestational diabetes, prediabetes, and borderline diabetes). Hypertension was assessed separately, but with the same question format. Possible responses to these questions were yes, no, don’t know/not sure, or refused. Responses to questions in the BRFSS core for the two additional underlying conditions, arthritis and high cholesterol, were not analyzed because they have not been associated with increased risk for severe COVID-19 illness. Respondents giving positive answers to 1) a heart attack/myocardial infarction or 2) angina or coronary heart disease were counted as having coronary heart disease.

§ California, Connecticut, Delaware, Florida, Georgia, Hawaii, Idaho, Illinois, Kansas, Louisiana, Maryland, Massachusetts, Michigan, Minnesota, Mississippi, Missouri, Montana, Nebraska, New Hampshire, New Jersey, New Mexico, New York, North Carolina, North Dakota, Pennsylvania, Rhode Island, South Carolina, Tennessee, Texas, Washington, and Wisconsin.

¶ Active duty military respondents were not included in the analyses.

** Industry was elicited by the question “What kind of business or industry do you work in? For example, hospital, elementary school, clothing manufacturing, restaurant.” Occupation was elicited by the question “What kind of work do you do? For example, registered nurse, janitor, cashier, auto mechanic.”

†† https://www.census.gov/programs-surveys/cps/technical-documentation/methodology/industry-and-occupation-classification.html.

§§ https://www.cdc.gov/brfss/annual_data/2017/pdf/2017-response-rates-table-508.pdf.

¶¶ https://www.cdc.gov/brfss/annual_data/2018/pdf/2018-response-rates-table-508.pdf.

*** Minimum sample size for each occupation or industry was 1,000 in the analytic data set, selected to ensure reasonably narrow confidence limits for prevalence estimates and to meet reportability criteria for uncommon outcomes.

††† https://www.cdc.gov/coronavirus/2019-ncov/community/worker-safety-support/index.html.
